# Oral cavity and lip cancer: United Kingdom National Multidisciplinary Guidelines

**DOI:** 10.1017/S0022215116000499

**Published:** 2016-05

**Authors:** C Kerawala, T Roques, J-P Jeannon, B Bisase

**Affiliations:** 1 Head and Neck Unit, The Royal Marsden Hospital, London, UK; 2 Norfolk and NorwichUniversity Hospitals NHS Foundation Trust, Norwich, UK; 3 Department of Otolaryngology-Head and Neck Surgery, Guy's and St Thomas’ NHS Foundation Hospital Trust, King's College, London, UK; 4 Queen Victoria Hospital, East Grinstead, UK

## Abstract

This is the official guideline endorsed by the specialty associations involved in the care of head and neck cancer patients in the UK. It provides recommendations on the assessment and management of patients with cancer of the oral cavity and the lip.

**Recommendations:**

• Surgery remains the mainstay of management for oral cavity tumours. (R)

• Tumour resection should be performed with a clinical clearance of 1 cm vital structures permitting. (R)

• Elective neck treatment should be offered for all oral cavity tumours. (R)

• Adjuvant radiochemotherapy in the presence of advanced neck disease or positive margins improves control rates. (R)

• Early stage lip cancer can be treated equally well by surgery or radiation therapy. (R)

## Introduction

In order of decreasing frequency, malignant tumours of the oral cavity affect the anterior two-thirds of the tongue, floor of mouth, buccal mucosa, retromolar trigone, hard palate and gingivae. Tumours of the lip require separate consideration as their natural history differs from oral cavity disease. The overwhelming majority of oral cavity cancers are squamous cell carcinomas (SCCs). Non-squamous cell tumours are predominantly of salivary gland origin and are discussed elsewhere in these guidelines. The heterogeneous nature of oral cavity tumours, the functional and cosmetic sequelae of their management and the frequent medical co-morbidities that co-exist in this patient group demand that treatment options should be considered by a multidisciplinary team before reaching a final plan through consensus with the patient and carers. The overall treatment intention, whether curative or palliative, should be clearly communicated at the outset.

## Pathology

### Oral cavity

Carcinoma of the oral cavity may develop *de novo* or from a pre-malignant dysplastic lesion that appears clinically as leukoplakia, erythroplakia or a combination of the two. In both instances, chronic exposure to carcinogens such as tobacco or alcohol is thought to be important. Carcinogenesis is a multistep process that involves over expression of oncogenes and inactivation of tumour suppressor genes. The p53 suppressor gene has been identified as being important in oral cavity carcinomas in smokers. The presence of human papilloma virus (HPV) that expresses the p16 oncoprotein in oral cavity carcinoma in non-smokers is of significant importance as the cancers tend to occur in younger patients. However, HPV-related disease does not appear as frequently in the oral cavity as it does in the oropharynx and appears not to proffer as much of an improvement in prognosis.^1^ The importance of epidermal growth factor receptor (EGFR) status in oral cavity carcinoma remains unclear. Whilst over expression does appear to be related to poor prognosis, EGFR status does not yet appear to be correlated with response to targeted molecular therapies such as cetuximab.

Within the diagnosis of oral cavity SCC, several histological subtypes exist with different prognoses such as verrucous (better prognosis) and basaloid (worse prognosis) carcinomas. Oral SCCs are classified according to grade depending on several histopathological features such as degree of keratinisation, nuclear pleomorphism, cellular atypia and mitotic activity. They are divided into well, moderate and poorly differentiated carcinomas. However, tumour grade is of limited prognostic value due to the heterogeneity within a tumour and sampling error. Several other histopathological factors have been shown to be of prognostic importance such as tumour thickness, extra-capsular spread (ECS) of nodal metastasis^2^ and patterns of invasion. Oral tongue SCC of greater than 4 mm tumour thickness is considered to represent a >20 per cent risk of cervical lymph node metastatic involvement.^3^ Extra-capsular spread in cervical lymph nodes is consistently associated with an increased risk of local regional recurrence, distant metastasis and decreased survival. The pattern of invasion in oral SCC appears to be important in determining prognosis in that those cancers that have a non-cohesive invasive front and/or peri-neurial invasion appear to be associated with an increased risk of loco-regional relapse.^4^ These pathological factors therefore supplement the tumour–node–metastasis classification and are now incorporated in pathological datasets.

### Lip

Cancer of the lip is the most common malignant tumour affecting the head and neck. Its clinical behaviour is similar to that of skin cancer. Incidence rates are around 13.5 per 100 000 in Oceania, 12 per 100 000 in Europe and 12.7 per 100 000 in North America.^5^ The factors commonly cited as important in lip cancer are solar radiation, tobacco smoking and viruses. About 90 per cent of tumours arise in the lower lip with 7 per cent occurring in the upper lip and 3 per cent at the oral commissure.

Squamous cell carcinoma is the commonest histological tumour type in lip cancers, followed by basal cell carcinoma. The most common non-mucosal form of lip cancer arises from tumours of the minor salivary glands, with in converse to mucosal lip cancer the upper lip being more commonly involved than the lower.

## Clinical presentation

The majority of SCCs (>95 per cent) of the oral cavity are presented as ulcers or masses. Early lesions can be subtle and appear as flat, discoloured areas (leukoplakia or erythroplakia).^6^ A non-healing ulcer is the most common presentation. Advanced tumours can present with invasion of neighbouring structures causing tooth mobility, trismus, sensory change, referred otalgia and neck masses. The clinical presentation of cancer of the lip is usually that of an exophytic, crusted lesion with variable invasion into underlying muscle (related to the size of the primary tumour). The adjacent lip often shows features of actinic sun damage such as colour change, mucosal thinning and various associated areas of leukoplakia.^7^

## Assessment and staging

### Clinical examination

Clinical examination is useful in identifying new tumours and for surveillance after treatment. Given its importance in diagnosis and treatment planning, a systematic approach must be adopted to include the primary site and neck, with assessment of the index tumour size as well as any potential invasion of local structures. The examination should be preceded by a focused history to elucidate any potential co-morbidities and social circumstances that may influence the choice of treatment.

### Imaging considerations

Imaging of early stage tumours of the lip is usually not indicated. However, advanced tumours of the lip (particularly if they are adherent to the adjacent mandible) require computed tomography (CT) or magnetic resonance imaging to allow complete staging and treatment planning with regard to resection margins which may of necessity include adjacent bone.

Oral cavity tumours are almost always staged with cross-sectional imaging to include the chest where the demonstration of simultaneous pulmonary parenchymal disease may influence curability.^8^^,^^9^ Sentinel node lymph node biopsy has been shown to be an effective method of assessment of the neck in early stage oral cancers.^10^

### Pre-treatment staging

Staging of primary cancer of the lip and oral cavity is similar and shown in Table I. T4 tumours of the lip usually only invade the anterior mandible or maxilla rather than other structures.

**Table I:** T Staging for oral cavity tumours

TX	Primary tumour cannot be assessed
T0	No evidence of primary tumour
Tis	Carcinoma in situ
T1	Tumour 2 cm or smaller in greatest dimension
T2	Tumour larger than 2 cm but 4 cm or smaller in greatest dimension
T3	Tumour larger than 4 cm in greatest dimension
T4a	Tumour invades the larynx, deep/extrinsic muscle of tongue, medial pterygoid, hard palate or mandible
T4b	Tumour invades lateral pterygoid muscle, pterygoid plates, lateral nasopharynx or skull base or encases carotid artery

## Management

### Oral cavity

Although there is no randomised data exclusively comparing the different treatment modalities available in the management of oral cavity cancer, non-surgical clinical trials often present this subsite in combination with others in the head and neck. Two-year crude survival rates are around 85 per cent for stage I disease, 70 per cent for stage II disease^11^, 50 per cent for stage III disease and 40 per cent for stage IV disease.^12^

#### General principles

##### Surgery

Factors such as fitness for anaesthesia, previous cancer treatment and patient choice as well as the skill mix and resources available to the treating team must be considered.^13^^,^^14^ There are a number of different options available under the broad banner of surgery: conventional surgery, laser surgery, thermal surgery and photodynamic therapy (PDT).^15^ Curative surgery for cancer of the oral cavity involves resection of tumour with an appropriate safety margin and subsequent reconstruction of the tissues in order to maintain function. The size and location of the primary tumour determine the need or otherwise for adjuncts such as temporary tracheostomy and access procedures. Many tumours in the anterior aspect of the oral cavity can be accessed via the transoral route. This is ideal, since in so doing the circumferential muscular sphincter is maintained and scars avoided. However, as tumours increase in size and become more posteriorly placed, a controlled resection may only be possible by performing either a lingual release or resorting to lip-split and mandibulotomy. There are several options for the lip skin incision with some form of *Z*-plasty being desirable to both disguise and lengthen the scar, thus preventing post-operative wound contraction and distortion to the vermilion border.

Effective tumour ablation is achieved by ensuring good visibility which in turn is dependent on appropriate access. In order to maximise the chances of achieving complete tumour resection with a clear margin of normal tissue, both visual inspection and palpation must be employed. The method of ablation, be it scalpel, laser, diathermy or coblation, is a matter of personal preference. For small, superficial lesions laser vaporisation may be employed although this often does not permit accurate histological assessment of the adequacy of resection and so may compromise decisions surrounding the need or otherwise for adjuvant treatments. Lasers and thermal techniques, whilst reducing the amount of intra-operative bleeding, can cause histological artefact and morphological distortion of tissue margins. Coblation involves the generation of bipolar radio-frequency waves. Tissue temperatures of around 60 °C ensue, much lower than temperatures generated by conventional diathermy. Although this is claimed to reduce post-operative pain, the technique has been associated with increased levels of haemorrhage in certain head and neck sites.

The primary aim of surgery in oral cavity cancer is tumour resection with a clinical clearance of ideally 1 cm (vital structures permitting). ‘Close’ margins (defined as a histopathological margin of less than 5 mm) mean further surgery or adjuvant radiotherapy (RT) and should be discussed by the multidisciplinary team. The use of intra-operative frozen sections to assist marginal clearance is controversial.^16^ Although the accuracy is good in histological terms, they can give a false sense of security and invariably prolong operative time. Adoption of a Mohs-type technique where the whole of the resection bed is mapped out is impractical given the size of the average intra-oral resection. Intra-operative tumour tissue marking has been attempted with agents such as toluidine-blue but this has limited value in marginal clearance because of high false positive rates.^17^ Where bony resection is required, the assessment is largely based upon clinical and radiological findings.^18^ Intra-operative techniques such as periosteal stripping however remain reliable. Frozen section of cancellous bone can be used to guide the extent of the resection.

Cervical lymphadenectomy in the form of elective neck dissection offers improved overall and disease-free survival compared with therapeutic neck dissection for the majority of oral cancers with recent evidence suggesting advantages even for tumours less than 4 mm in thickness.^19^ Sentinel node lymph node biopsy may be indicated for small (T1 and T2) cancers since a negative sentinel node biopsy can avoid the morbidity of neck dissection and may be more cost-effective.^10^

**Recommendations**

• Surgery remains the mainstay of management for oral cavity tumours (R)
• Tumour resection should be performed with a clinical clearance of 1 cm vital structures permitting (R)
• Elective neck treatment should be offered for all oral cavity tumours (R)

##### Radiotherapy ± chemotherapy

In the oral cavity, primary radiochemotherapy is less commonly utilised than other head and neck sites. However, it should be considered in selected patients. Concurrent radiochemotherapy combines platinum-based chemotherapy with external beam radiotherapy (EBRT) to 70 Gy. While the most recognised concurrent chemotherapy regimen is cisplatin 100 mg/m^2^ three weekly, varying doses and schedules are acceptable practice, as is substitution by carboplatin. Patients undergoing radiochemotherapy require speech, swallow and dietetic support, in both the acute and long-term setting. Patients who are excluded from platinum-based chemotherapy may be considered for EBRT with cetuximab under National Institute for Health and Care Excellence guidance. Neo-adjuvant chemotherapy with taxanes, cisplatin and 5-fluro-uracil (TPF) is a potent combination in advanced, inoperable disease in fit patients, if followed by concurrent radiochemotherapy.

External beam radiotherapy is not usually recommended as the primary curative treatment in oral cavity tumours because the significant morbidity of treatment limits radiation dose and therefore cure rates. Severe mucositis of the treated volume during and immediately after treatment is inevitable and will affect function and nutrition. Long-term pain is a common sequelae if high enough radiation doses to cure primary tumours are used while osteoradionecrosis of the mandible is a particular risk when irradiating the oral cavity. External beam radiotherapy alone can be used to treat the neck prophylactically after excision of a small primary without a neck dissection. Brachytherapy as sole treatment or as a boost after EBRT can produce cure rates equivalent to those in surgical series. As the radiation dose is concentrated in the tumour tissue more effectively than with EBRT, higher doses and fewer long-term side effects can be achieved. Brachytherapy requires specific expertise which is not widely available in the UK.

Adjuvant RT improves local control and overall survival when added to surgery in locally advanced cancers. It should be considered in all patients with larger T3 or T4 tumours, where there is ECS or N2–3 neck disease. Other poor prognostic factors such as grade or peri-neurial invasion may also inform the decision.^4^ The morbidity of radiation to the primary site in the oral cavity means the benefits and side effects should be carefully considered with each individual patient.

Concomitant chemotherapy improves the effectiveness of adjuvant RT – more so in oral cavity tumours than in other primary sites of the upper aerodigestive tract – and should always be considered in patients over 71 years old with relevant histological features when RT is discussed.^20^ However, it increases the acute and late morbidity of treatment. In patients with incurable disease, a short course of palliative RT may help to improve local symptoms. Palliative chemotherapy with platinum-based drugs and 5FU or capecitabine can also be considered to help symptoms and improve survival.

#### Early stage cancer

Early stage tumours (T1 and small T2) can be adequately treated with either surgery or brachytherapy. Treatment choice may be influenced by tumour size, location, depth of invasion, proximity to bone, growth patterns including differentiation, neck nodal disease and access to services.

#### Advanced stage cancer

For advanced disease, stages III and IV (T3, T4 N0 and T1–4 N1), traditional management includes surgical resection, neck dissection, reconstruction and post-operative RT. The latter should be offered to at least 60 Gy equivalent and optimally start within 6 weeks of surgery. In fit patients under the age of 71, adjuvant radiochemotherapy up to 66 Gy with concurrent platinum-based chemotherapy should be considered for those with positive surgical margins and/or ECS.^21^

**Recommendation**

• Adjuvant radiochemotherapy in the presence of advanced neck disease or positive margins improves control rates (R)

#### Recurrent cancer

Patients with locally recurrent disease should be fully restaged and assessed for consideration of curative treatment in the form of repeat surgery, possible EBRT or brachytherapy if available. Palliative RT may be used, either over short fractionation schedules or split course, for patients with advanced and inoperable disease, or those who are not fit for a more toxic, radical approach. Palliative chemotherapy should be considered for inoperable, recurrent and or metastatic disease, when possible patients should be offered entry to clinical trials.

#### Reconstruction following surgical ablation of oral cavity tumours

There is a plethora of retrospective series reporting technique and outcome of a wide range of reconstructive techniques for the repair of defects following ablation for oral cavity tumours.^22^^,^^2^,^3^ However, there are no randomised controlled trials. The literature suffers from a wide range of heterogeneous factors introducing bias including tumour sites, stages, patient variables, operators, surgical techniques, study designs, small numbers, lack of clarity for treatment intention and the reporting of different outcome measures.

Reconstructive options include local flaps, regional pedicled flaps and microvascular free tissue transfer discussed elsewhere in the guidelines.^2^,^4^ Hard tissues may be reconstructed using free autologous bone grafts but more commonly involve the use of free tissue transfer from iliac crest, fibula, radius or scapula.

### Lip

#### General principles

Early stage cancer can be treated equally well by surgery or radiation therapy. The five-year crude survival rates for surgical treatment are about 75–80 per cent for T1 to T2 tumours, dropping to 40–50 per cent for T3 and T4 tumours. The primary lymphatic drainage of the lower lip is to submental and submandibular level cervical lymph nodes. Neck dissection is generally not performed in the absence of clinically suspicious cervical lymph nodes as more than 5 per cent of patients are likely to develop recurrence in the neck following treatment of the primary lesion. The presence of cervical nodes at presentation is a poor prognostic indicator. Small lesions are managed by simple surgical excision and primary closure. Equally good results can be achieved with fractionated EBRT or brachytherapy. External beam radiotherapy using electrons or orthovoltage photons minimises dose to the oral cavity so that mucositis occurs only on the treated lip.

Larger lesions of the lip require more consideration with regard to reconstruction techniques. The functional outcome of the repair with regard to lip sensitivity and muscle function also needs to be taken into consideration. Whenever possible full thickness skin flaps (skin, muscle and mucosa) should be used. The repair should provide sufficient mucosa contiguous to the commissure to avoid contracture. Superficial field change lesions affecting the external vermilion of the lip such as leukoplakia or actinic keratosis are best managed via a lip shave and mucosal advancement.

Various studies have shown that for small tumours radiation therapy can achieve a cure rate equivalent to that obtained surgically. However, the cosmetic results of EBRT to the lip are usually not as satisfactory as surgical excision and repair. Surgical excision of small lip tumours involves relatively minor surgery, often under local anaesthetic and may be therefore less burdensome for the patient than a course of RT. The lower lip is one of the few ideal sites for orthovoltage therapy. Using a single anterior field a fractionated course of 50 Gy in 15 fractions over 3 weeks is administered. Brachytherapy can produce good aesthetic results but is not widely available in the UK. Iridium^192^ can be used in the treatment of lip cancer. Patients can be treated twice a day for 4–5 days with a total radiation dose between 40 and 45 Gy in 8–10 fractions.

#### Lower lip

Small lesions invading into the adjacent muscle are amenable to a wedge excision. The excision can also be completed using a ‘W’ plasty or half ‘W’ plasty to avoid the inferior aspect of the excision encroaching on the crease line of the chin. If the dimensions of the lip resection require the introduction of tissue to minimise functional problems and microstomia, then this may be by means of Abbe, Abbe-Estlander or Karapandzic flaps. The Estlander modification of the cross-lip flap is used to reconstruct the oral commissure. The Karapandzic flap is useful for defects involving more than two-thirds of the lower lip, where the defect is in the midline. The main advantage of the Karapandzic flap is that the nerve and blood supply is retained and the underlying orbicularis muscle rotated so that a sensate functional lip reconstruction results. The various reconstructive options are identified in Table II. With larger defects of the lower lip reconstruction requires either large cheek flaps to be advanced to repair the defect or the use of free tissue transfer. The common forms of cheek flap include the bilateral Gillies fan flaps or the Bernard–Webster cheek flap reconstruction. Free tissue transfer is required for lip reconstruction when the total remaining lip or adjacent rotated tissue is insufficient to create a reasonable circular stoma.

**Recommendation**

• Early stage lip cancer can be treated equally well by surgery or radiation therapy (R)

**Table II:** Reconstructive options for lower lip defects

Defect size	Procedure
<1/2	Wedge excision
1/2 to 2/3	Karapandzic flap
	Abbe-Estlander flap
>2/3	Bernard Burow
	Gillies fan flap
	Webster flap
	Free flap

#### Upper lip

Similar to lower lip defects wedge excisions and advancement flaps can address upper lip defects which involve up to one half of the width of the upper lip. Care should be taken to respect the relevant aesthetic subunits. Defects of less than a third in the midline can be closed primarily. Defects involving greater than half of the lip can be reconstructed with cross-lip flaps from the lower lip. Peri-alar crescentic advancement flaps can be used to disguise the advancement of the upper lip when the advancement encroaches to the medial part of the nose. For defects involving more than two-thirds of the lip, a Burow-Diffenbach reconstruction can be performed. This flap replaces upper lip defects by utilisation of laterally based advancement flaps. Bilateral peri-alar crescentic excisions are required to provide adequate advancement. The various reconstructive options are identified in Table III.

**Table III:** Reconstructive options for upper lip defects

Defect size	Procedure
<1/2	Wedge excision
1/2–2/3	Peri-alar crescentic flap
	Reverse Karapandzic flap
	Abbe–Estlander flap
>2/3	Burow–Diffenbach flap
	Free flap

Most large series in the literature show that the majority of patients have small lesions without palpable cervical metastases although the incidence of synchronous cervical metastases increases as the size of the primary tumour increases. The local recurrence rate is low due to the relative ease of surgical excision. Even re-excision because of local failure leads to salvage in 75–80 per cent of cases.

## Developing therapeutic regimens

Neoadjuvant chemotherapy with TPF followed by surgery and then RT is accruing evidence in other primary sites. Radio chemotherapy with the addition of targeted agents requires further evaluation. Radiotherapy alone *vs* RT plus cetuximab in intermediate cancers and the use of positron emission tomography–computed tomography to define the gross tumour volume and to assess response to non-surgical treatments is the subject of ongoing research. Agents such as palifermin and amifostine are under investigation to reduce radiation toxicity but are not yet in routine use. Molecular mapping to determine the individualised, sub-clinical spread to inform the clinical target volume requires further evaluation. Likewise further work is required to establish the long-term quality of life, toxicity recognition, management and support in head and neck cancer patients receiving radiochemotherapy.

Xerostomia is one of the most unpleasant permanent complications from RT of the oral cavity. Sparing of the salivary glands by intensity-modulated radiation therapy may improve toxicity without reduction in local control.

The efficacy of hyperbaric oxygen in the prevention of osteoradionecrosis remains unproven, as does the use of medical therapies such as pentoxifylline and tocopherol in established cases.

Photodynamic therapy has been advocated as a technique which causes selective tumour destruction by cell apoptosis. Advocates suggest minimal scarring and preservation of uninvolved tissue thereby minimising any functional deficit caused by tumour ablation. Unfortunately the photograph-sensitising agents currently available are insufficiently selective to prevent normal tissue damage and patients must be protected from exposure to sunlight for several days. Since the wound sloughs and heals by secondary intention, there is little benefit in functional terms of PDT over the more traditional techniques. Foscan^®^ mediated PDT can also be used to treat primary cancer of the lip, where treatment yields complete response rates comparable with those published for surgery or RT. The lack of tissue memory for PDT means that unlike RT this treatment can be repeated on a number of occasions.

### Key points

• The majority of malignant tumours of the oral cavity are squamous cell carcinomas
• The clinical behaviour of lip cancer is akin to skin cancer
• While tobacco and alcohol are the main carcinogens implicated in oral cavity cancer, a small but significant role for human papilloma virus is recognised
• Surgical resection is the primary modality used to manage most oral cancers
• Elective neck management is indicated for any tumour when the risk of occult nodal involvement is >20 per cent
• Several reconstructive options exist to repair soft tissue and bony defects after tumour resection
• Tumour thickness, positive margins and extra-capsular spread of nodal metastasis and pattern of invasion have been shown to have significant prognostic value
• Post-operative adjuvant radiation or radiochemotherapy should be considered in the presence of unfavourable disease factors.
